# Effects of norepinephrine transporter gene variants on NET binding in ADHD and healthy controls investigated by PET

**DOI:** 10.1002/hbm.23071

**Published:** 2015-12-17

**Authors:** Helen L. Sigurdardottir, Georg S. Kranz, Christina Rami‐Mark, Gregory M. James, Thomas Vanicek, Gregor Gryglewski, Alexander Kautzky, Marius Hienert, Tatjana Traub‐Weidinger, Markus Mitterhauser, Wolfgang Wadsak, Marcus Hacker, Dan Rujescu, Siegfried Kasper, Rupert Lanzenberger

**Affiliations:** ^1^ Department of Psychiatry and Psychotherapy Medical University of Vienna Vienna Austria; ^2^ Department of Biomedical Imaging and Image‐Guided Therapy Division of Nuclear Medicine, Medical University of Vienna Vienna Austria; ^3^ Department of Psychiatry University of Halle Halle Germany

**Keywords:** norepinephrine transporter, positron emission tomography, single nucleotide polymorphisms, neuroimaging genetics, attention deficit hyperactivity disorder

## Abstract

Attention deficit hyperactivity disorder (ADHD) is a heterogeneous disorder with a strong genetic component. The norepinephrine transporter (NET) is a key target for ADHD treatment and the NET gene has been of high interest as a possible modulator of ADHD pathophysiology. Therefore, we conducted an imaging genetics study to examine possible effects of single nucleotide polymorphisms (SNPs) within the NET gene on NET nondisplaceable binding potential (BP_ND_) in patients with ADHD and healthy controls (HCs). Twenty adult patients with ADHD and 20 HCs underwent (*S,S*)*‐*[^18^F]FMeNER‐D_2_ positron emission tomography (PET) and were genotyped on a MassARRAY MALDI‐TOF platform using the Sequenom iPLEX assay. Linear mixed models analyses revealed a genotype‐dependent difference in NET BP_ND_ between groups in the thalamus and cerebellum. In the thalamus, a functional promoter SNP (−3081 A/T) and a 5′‐untranslated region (5′UTR) SNP (−182 T/C), showed higher binding in ADHD patients compared to HCs depending on the major allele. Furthermore, we detected an effect of genotype in HCs, with major allele carriers having lower binding. In contrast, for two 3′UTR SNPs (*269 T/C, *417 A/T), ADHD subjects had lower binding in the cerebellum compared to HCs depending on the major allele. Additionally, symptoms of hyperactivity and impulsivity correlated with NET BP_ND_ in the cerebellum depending on genotype. Symptoms correlated positively with cerebellar NET BP_ND_ for the major allele, while symptoms correlated negatively to NET BP_ND_ in minor allele carriers. Our findings support the role of genetic influence of the NE system on NET binding to be pertubated in ADHD. *Hum Brain Mapp 37:884–895, 2016*. © **2015 The Authors Human Brain Mapping Published by Wiley Periodicals, Inc**.

## INTRODUCTION

Attention deficit hyperactivity disorder (ADHD) is the most frequent neurodevelopmental disorder diagnosed in children. It is characterized by inattention, hyperactivity, and impulsiveness which frequently leads to severe social, academic, and vocational dysfunction [De La Fuente et al., 2013]. In around 30% of ADHD cases, the symptoms persist through adolescence into adulthood [Barbaresi et al., 2013]. Symptoms differ in adults compared to children, such as hyperactivity decreases while problems with inattention persist [Volkow and Swanson, 2013]. ADHD has a strong genetic component with a heritability estimated to be around 0.77 [Curatolo et al., 2009]. Though the heritability is rather high in ADHD, studies have failed to indicate a single gene responsible for the course of ADHD, suggesting complex polygenetic mechanisms and gene environment interactions to be of importance [Banaschewski et al., 2010].

Norepinephrine (NE) neurotransmission has been hypothesized to be altered in various disorders, such as depression, PTSD, Alzheimer's disease, and ADHD [Biederman and Spencer, 1999; Gulyas et al., 2010; Klimek et al., 1997; Pietrzak et al., 2013]. NE has long been discussed to be dysregulated in ADHD since frequently prescribed psychopharmaca such as methylphenidate (MPH) and atomoxetine (ATX) target the dopaminergic and NE systems by increasing the extracellular neurotransmitter levels through inhibition of the respective reuptake transporters [Hannestad et al., 2010; Logan et al., 2007]. MPH and ATX, a selective NE reuptake inhibitor, have been proven clinically effective in improving core symptoms in ADHD [Asherson et al., 2014], although up to 40% of patients being ascribed to stimulant and nonstimulant medication do not respond [Newcorn et al., 2008, 2009]. Recently, guanfacine, an alpha‐2 adrenergic receptor agonist, has also been used as an effective treatment option for patients with ADHD [Newcorn et al., 2013]. It is, therefore, likely that alterations in the NE system may predispose to ADHD and thus, the norepinephrine transporter (NET) gene is suspected to play a major role in ADHD pathogenesis. The gene encoding for the NET (SLC6A2) contains certain single nucleotide polymorphisms (SNPs) that have been investigated in pathological conditions [Hahn and Blakely, 2007]. In association and linkage studies, various SNPs have been found to be involved throughout the ADHD population [Kim et al., 2006a; Sengupta et al., 2012]. Results, however, have varied, and there is some contradictory results confounding this theory [Barr et al., 2002; Xu et al., 2005].

As for in vivo brain quantification of the NET, specifically in ADHD, literature is quite scarce until now. In a recently published study, our group demonstrated no differences in NET nondisplaceable binding potential (BP_ND_) in patients with ADHD compared to healthy controls (HCs) [Vanicek et al., 2014]. It is of high interest to examine whether genetic variants in the NE system have an effect on NET BP_ND_ which could shed light on individual differences in susceptibility to ADHD. Additionally, to the best of our knowledge, no positron emission tomography (PET) study is available so far investigating polymorphisms in the NE system on the NET binding, neither in HCs nor in patients with ADHD.

Thus, the aim was to examine the relationship between the effects of SNPs in the NE system and the NET BP_ND_ in a cohort comprising of ADHD subjects and HCs matched for age and sex. We hypothesized that ADHD subjects carrying either major or minor alleles will have higher binding compared to their healthy matched controls. High binding subcortical regions believed to be principal areas in behavioral and attentional control were selected [Arnsten and Rubia, 2012], whereas cortical regions were dismissed due to the defluorination and bone spill over of the radioligand (*S,S*)*‐*[^18^F]FMeNER‐D_2_. Moreover, we hypothesized that the symptoms of hyperactivity and impulsivity would correlate to with NET BP_ND_ in areas related to motoric activity (putamen, cerebellum, midbrain) whilst symptoms of inattention would correlate with NET BP_ND_ in the thalamus.

## MATERIALS AND METHODS

### Subjects

Twenty adult ADHD patients (age ± SD: 30.8 ± 10.9, 14 males) and 20 HCs (age ± SD: 30.4 ± 10.9, 14 males) were recruited through ADHD outpatient clinic at the Department of Psychiatry and Psychotherapy, Medical University of Vienna, and from the local community via advertisement as previously published elsewhere [Vanicek et al., 2014]. All patients had been free from psychopharmacological treatment for at least 6 months prior to screening visit. During the prescreening, medical examinations including withdrawal of blood samples were performed to ensure physical well being of participants. All participants underwent a multidrug urine test to assess current substance abuse. For inclusion, patients had to have a current ADHD diagnosis as well as a history of childhood ADHD. Five of the 20 patients had their first diagnosis in childhood. Subjects were interviewed using the Conners' Adult ADHD Diagnostic Interview for DSM‐IV (CAADID, Conners, 1999), Conners' Adult ADHD Rating Scale Investigater‐Screen Version (CAARS‐Inv:SV), Conners' Adult ADHD Rating Scale: Observer‐Screen Version (CAARS‐O:SV) and the Conners‘Adult ADHD Rating Scale: The self‐report screening Version (CAARS‐S:SV). To exclude any current comorbidities, subjects were interviewed using the Structural Clinical Interview for DSM‐IV Axes I and II disorders. HCs were naïve to psychopharmacological treatment. Participants signed written consent forms for the study and were reimbursed financially for their participation. The study was approved by the Ethics committee of the Medical University of Vienna.

### Selection of Single Nucleotide Polymorphisms

**Figure 1:**
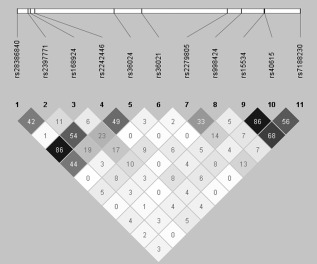
Linkage disequilibrium plot of single nucleotide polymorphisms (SNPs) considered for inclusion. Depicted are 11 genotyped SNPs and the pairwise *R* ^2^ between them. At the top are the relative positions of the SNPs to one another on the NET gene. Below are the rs numbers for each corresponding SNP and the color scheme shows the strength of the of their *R* ^2^ value. White= *R* ^2^= 0, shades of gray = 0 < *R* ^2^ < 1 and black = *R* ^2^ = 1.

Eleven SNPs (Fig. 1) were considered for inclusion, which were selected upon previous association studies [Bobb et al., 2005; Sengupta, et al., 2012; Thakur et al., 2012] and functional effect studies [e.g., functional promoter −3081 A/T (rs28386840), where the minor allele (T) has been shown to decrease promoter activity] [Kim et al., 2006a]. Three SNPs were included in genotyping to extend the 3′ flanking region (rs15534, rs40615, rs7188230). Furthermore, three SNPs were chosen to extend the 5′ region of NET (rs2397771, rs168924, rs2242246). Haploview version 4.2 (http://www.broad.mit.edu/mpg/haploview/) was used to test whether frequencies were according to Hardy–Weinberg equilibrium. Furthermore, the tag function in Haploview was used to identify SNPs in high‐linkage disequilibrium. It refers to the nonrandom association of alleles at different loci, allowing an identification of genetic variations by the information derived from one or more SNPs [Hu et al., 2004; Stram, 2004]. Thus, these tagged SNPs were used for further analysis. For final analysis, the following four SNPs were used as identified using the tag function in Haploview: −3081 A/T (rs28386840) and −182 T/C (rs2242446) (*r* ^2^ = 869), and *269 T/C (rs15534) and *417 A/T (rs40615) (*r* ^2^ = 0.866). For simplicity's sake, they will be referred to by their rs number in this article.

### Genotyping

Procedures were preformed as previously described [Baldinger et al., 2014]. In short, 9 ml. EthyleneDiamineTetraacetic Acid (EDTA) blood samples were drawn from each subject and DNA was isolated from whole blood using the QiaAmp DNA blood maxi kit (Qiagen, Hilden, Germany). Genotyping was performed using the iPLEX assay on the MassARRAY MALDI‐TOF mass spectrometer as described [Oeth et al., 2009]. Allele specific extension products were identified and genotypes allocated by Typer 3.4 Software (Sequenom, San Diego, CA). All applied quality criteria were met [individual call rate >80%, SNP call rate >99%, identity of genotyped of CEU trios (Coriell Institute for Medical research, Camden, NJ) with HapMap database >99%].

### Positron Emission Tomography

Scans were conducted at the Department of Biomedical and Image‐guided Therapy, Division of Nuclear Medicine at the Medical University of Vienna. Each subject underwent a PET (General Electric Medial Systems, Milwaukee, WI) scan using the tracer (*S,S*)*‐*[^18^F]FMeNER‐D_2_, synthesized as previously described [Rami‐Mark et al., 2013]. (*S,S*)*‐*[^18^F]FMeNER‐D_2_ is currently the most suitable radioligand for in vivo NET quantification previously described [Vanicek et al., 2014]. Briefly, fluorine‐18‐labelled reboxetine analogue allows, due to its long half‐life (*t*_1/2_ = 109.77 min) and excellent affinity and selectivity, to reach the specific binding equilibrium within the time‐frame of the PET measurement. A 5‐min transmission scan using a retractable ^68^Ge rod sources for tissue attenuation correction was performed prior to the dynamic emission scan acquired in 3‐D mode. Data acquisition started 120 min after a bolus i.v. injection of 4.7 MBq/kg body weight (ADHD patients: 393 ± 95 MBq, HC: 384 ± 61 MBq; *P* > 0.05, *t*‐test) of (*S,S*)‐[^18^F]FMeNER‐D_2_. Mean specific radioactivity of (*S,S*)‐[^18^F]FMeNER‐D_2_ was 537 ± 383 GBq/μmol (ADHD patients) and 473 ± 218 GBq/μmol (HC) (*P* >0.05, *t*‐test). Brain radioactivity was measured in a series of six consecutive time frames lasting 10 min each in the interval of 120–180 min after tracer bolus application. Acquired data were reconstructed in volumes consisting of 35 transaxial sections (128 × 128 matrix) using an iterative filtered back projection algorithm (FORE‐ITER) with a spatial resolution of 4.36‐mm full‐width at half maximum 1 cm next to the center of the field of view. For coregistration, magnetic resonance (MR) images were acquired from all participants on a 3‐Tesla Philips scanner (Achieva) using a 3‐D T_1_ FFE‐weighted sequence, yielding 0.88‐mm slice thickness and in plane resolution of 0.8 × 0.8 mm [Vanicek et al., 2014].

### Data Preprocessing and Quantification of NET

As described previously [Vanicek et al., 2014], each time frame of the dynamic PET scan was realigned to the mean of frames with no head motion, which was identified by visual inspection. These summed realigned images were then coregistered to each individual's MRI scan using a mutual information algorithm in SPM8 (Wellcome Trust Centre for Neuroimaging, London, UK: http://www.fil.ion.ucl.ac.uk/spm/). Parametric images of NET BP_ND_ were computed using the caudate as the reference region. The quantification was done as previously described [Arakawa et al., 2008]. Briefly, the ratio method was used to express the BP_ND_ as area under the time‐activity curve of the target region/area under the time‐activity curve for the reference region. The ratio method was highly correlated to the golden standard used in their study, values were *r* = 0.88 (*y* = 0.71*x* + 0.29) in the thalamus and *r* = 0.88 (*y* = 0.86*x* + 0.12) for other brain regions. The integration interval of 120–180 min was used. Manual delineation of the caudate ROI was performed on individual MR images using PMOD image analysis software, version 3.1 (PMOD Technologies, Zurich, Switzerland, http://www.pmod.com). MRI scans were spatially normalized using SPM8 (Wellcome Trust Centre for Neuroimaging, London, UK; http://www.fil.ion.ucl.ac.uk/spm/) and the resulting transformation matrices applied to the coregistered parametric images warping them into MNI standard space.

### Regions of Interest

Four regions of interest (ROIs) were selected including NET rich regions [Schou et al., 2005] as well as regions thought to be “core" regions in behavioral control (inattention, impulsivity, hyperactivity) [Arnsten and Rubia, 2012]. These were the thalamus, midbrain with pons (including the locus coeruleus), putamen, and cerebellum. Cortical regions, such as the prefrontal cortex (PFC) were not taken into account due to the bone spill over of (*S,S*)*‐*[^18^F]FMeNER‐D_2_ inherent to the radioligand. NET BP_ND_ for each region was extracted from parametric maps from the Hammers Maximum Probability Atlas [Hammers et al., 2003].

### Statistical Analysis

Descriptive statistics were computed and regional NET BP_ND_ values were evaluated for normality using the Shapiro–Wilk test. For each analysis, subject were grouped according to their genotype, that is, minor allele carriers versus major allele homozygotes. Genotype frequencies were determined and found to be distributed according to the Hardy–Weinberg equilibrium (*P* > 0.1).

To examine the effect of genotypes on NET BP_ND_, linear mixed models for each SNP were computed, using the genotype (homozygous major vs. minor allele carriers) and group (ADHD patients vs. HC) as fixed factors and ROI as a repeated factor and the NET BP_ND_ as the dependent variable. Possible effects of cofactors (age and sex) were also tested for and were excluded if insignificant.

A separate model for each SNP was computed as follows; linear mixed model with the factors group, ROI, and genotype as the independent variables and the BP_ND_ as the dependent variable. For each model, main effects were tested for, and interactions among ROI, group, and genotype. If rendered significant, further analysis included testing for interaction between group and genotype, separated by ROI. Further analysis included post hoc *t‐*tests.

The model prevailing the best fit was the autoregressive 1 (AR(1)). Individual slopes and intercepts were fitted for subjects and for random effects the variance components structure was used.

To test whether there was any effect of behavioural subscales on NET BP_ND_ depending on genotypes, Pearson's correlation coefficient was used. All analyses were computed using SPSS version 22.0 (IBM Corp. Released 2013. IBM SPSS Statistics for Windows, Armonk, NY: IBM Corp). Each model was corrected for multiple comparisons using the false discovery rate (FDR) at a significance level of *α* = 0.05 [Benjamini et al., 2001].

## RESULTS

Demographics and allele counts of study subjects can be seen in Table 1. Control and ADHD groups did not differ significantly in terms of age and sex.

**Table 1:** Demographics, psychological tests, past comorbidities, and allele frequencies between patients with ADHD and HCs

			Controls (*n* = 20)	ADHD (*n* = 20)
	Age		30.4 ± 10.9	30.8 ± 10.9
	Sex	M/F	14/6	14/6
SNP	rs28386840	A/T	9/10	14/6
SNP	rs2242446	T/C	9/9	12/8
SNP	rs15534	C/T	13/7	11/9
SNP	rs40615	T/A	12/8	10/10
CAARS	Total score		0.32 ± 0.82*	37.45 ± 8.23*
CAARS	Hyperactive/Impulsive		0.21 ± 0.63*	19.45 ± 5.89*
CAARS	Inattention		0.11 ± 0.32*	18 ± 4.78*
	Past comorbidities			
	Depression			*n* =7
	Drug abuse			*n* = 2

Significant differences between groups are indicated with * at *P* < 0.001. Genotype frequencies are shown for major/minor allele

**Figure 2:**
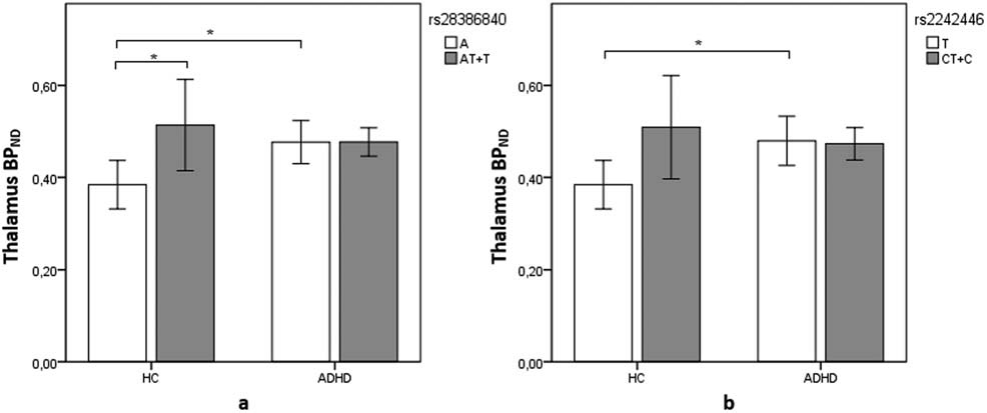
Differences in NET BP_ND_ in the thalamus between alleles rs28386840 (−3081 A/T) (**a**) and rs2242446 (−182 T/C) (**b**). The white bars depict the major alleles while the gray ones depict the minor alleles. Major allele (A) carriers for rs28386840 were (*n* = 9) for HCs and (*n* = 14) for ADHD subjects. Minor allele (T) carriers for rs28386840 were (*n* = 10) for controls and (*n* = 6) for ADHD subjects. Major allele (T) carriers for rs2242446 were (*n* = 9) for HCs and (*n* = 9) for ADHD subjects. Minor allele (T) carriers for rs2242446 were (*n* = 12) for controls and (*n* = 8) for ADHD subjects. Error bars indicate 95% confidence interval. Difference between groups is marked with an * in which the difference is at *P* < 0.05 corrected level significance.

For the functional promoter SNP (rs28386840) a significant three‐way interaction was detected between ROI, status and genotype (*F*_3.08_ = 104.6, *P* = 0.002, *P* < 0.05, corrected). On a ROI‐based level, a further analysis detected an interaction between status and genotype in the thalamus (*F*_11.16_ = 34.87, *P* = 0.002, *P* < 0.05, corrected). Post hoc *t*‐tests revealed that ADHD subjects had higher NET BP_ND_ than controls for the major allele (A) (*t* = −3.5, *P* = 0.006, *P* < 0.05, corrected) and no difference was detected for the minor allele (T) between groups (*t* = 0.73, *P* > 0.05). This is likely due to the difference in HCs between major and minor allele groups, with major allele having lower binding than the minor allele group (*t* = −3.06, *P* = 0.007, *P* < 0.05, corrected) (Table 2 and Fig. 2a).

**Table 2:** Linear mixed model effects summary for the SNP rs28386840

	Model rs28386840		
Fixed effects	df	*F* value	*P* value
Intercept	12.78	864.80	<.000
Group	26.34	0.70	0.41
ROI	83.66	195.59	<.000
rs28386840	39.97	1.77	0.19
group*ROI*rs28386840	104.60	3.08	0.002
*Separated by ROI*			
Cerebellum group*rs28386840	33.27	0.74	0.20
Midbrain group*rs28386840	34.31	4.10	0.049
Putamen group*rs28386840	32.97	1.16	0.29
Thalamus group*rs28386840	34.87	11.16	0.002

Values given are degrees of freedom (df), *F* values, and *P* values

For rs2242446, three‐way interaction was detected among ROI, group, and genotype (*F*_2.90_ = 103.57, *P* = 0.003, *P* < 0.05, corrected). Based on different ROIs, the analysis demonstrated an interaction between group and genotype in the thalamus (*F*_10.05_ = 33.90, *P* = 0.003, *P* < 0.05, corrected). Post hoc *t‐*test revealed that ADHD subjects had higher binding for the major allele (T) (*t* = −3.0, *P* = 0.008, *P* < 0.05, corrected) than controls and no difference was detected for minor allele (C) between groups (Table 3 and Fig. 2b). Which is likely due to difference in HCs between major and minor allele groups, which did not survive corrections (*t* = −2.54, *P* = 0.022, *P* > 0.05, corrected).

**Table 3:** Linear mixed model effects summary for the SNP rs2242446

	Model rs2242446		
Fixed effects	df	F value	*P* value
Intercept	9.10	930.46	<.000
group	20.97	0.42	0.52
ROI	80.45	200.78	<.000
rs2242446	32.83	1.61	0.21
group*ROI*rs2242446	103.57	2.90	0.003
*Separated by ROI*			
Cerebellum group*rs2242446	31.50	0.57	0.46
Midbrain group*rs2242446	34.66	6.47	0.026
Putamen group*rs2242446	32.12	1.41	0.24
Thalamus group*rs2242446	33.90	10.05	0.003

Values given are degrees of freedom (df), *F* values, and *P* values

**Figure 3:**
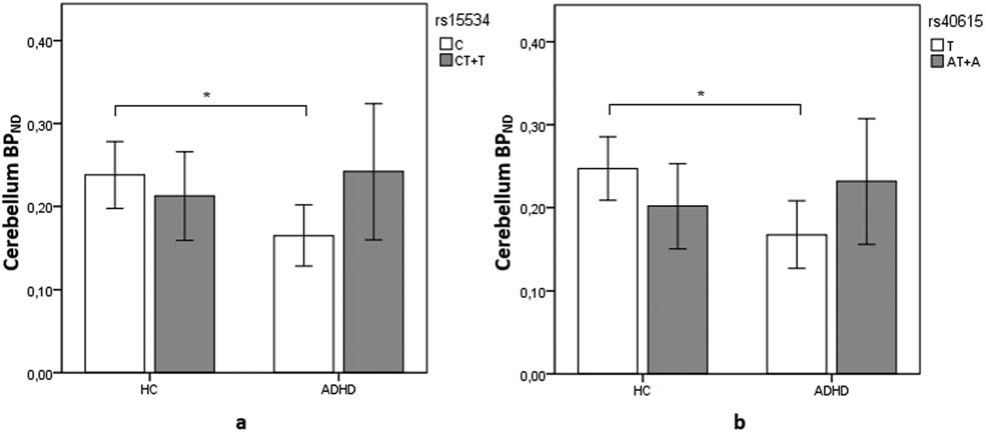
Differences in NET BP_ND_ in the cerebellum between alleles rs15534 (*269 T/C) (**a**) and rs40615 (*417 A/T) (**b**). The white bars depict the major alleles while the gray ones depict the minor alleles. Major allele (C) carriers for rs15534 were (*n* = 13) for HCs and (*n* = 11) for ADHD subjects. Minor allele (T) carriers for rs15534 were (*n* = 7) for controls and (*n* = 9) for ADHD subjects. Major allele (T) carriers for rs40615 were (*n* = 12) for HCs and (*n* = 10) for ADHD subjects. Minor allele (A) carriers for rs40615 were (*n* = 8) for controls and (*n* = 10) for ADHD subjects. Error bars indicate 95% confidence interval. Difference between groups is marked with an * in which the difference is at *P* < 0.05 corrected level significance.

A three‐way significant interaction was detected among rs15534 genotype, group, and ROIs (*F*_2.75_ = 117.52, *P* = 0.004, *P* < 0.05, corrected). After separating the analysis by each ROI to determine where the difference was, an interaction was detected between rs15534 genotypes and group in the cerebellum (*F*_7.73_ = 35.63, *P* = 0.009, *P* < 0.05, corrected). Post hoc *t‐*test revealed that controls carrying the major allele (C) in rs15534 had higher binding compared to major allele carrying patients (*t* = 3.19, *P* = 0.004, *P* < 0.05, corrected) (Table 4 and Fig. 3a). No difference was detected between minor allele (T) groups, and a trend between patients was detected between major and minor allele groups (*t* = 2.09, *P* = 0.051) and between minor and major allele in HCs (*t* = 1.80, *P* = 0.088).

**Table 4:** Linear mixed model effects summary for the SNP rs15534

	Model rs15534		
Fixed effects	df	*F* value	*P* value
Intercept	16.92	733.19	<.000
group	33.25	0.29	0.59
ROI	88.09	194.08	<.000
rs15534	51.62	0.61	0.44
group*ROI*rs15534	117.52	2.75	0.004
*Separated by ROI*			
Cerebellum group*rs15534	35.63	7.73	0.009
Midbrain group*rs15534	35.97	0.48	0.56
Putamen group*rs15534	35.62	2.78	0.89
Thalamus group*rs15534	33.49	0.95	0.34

Values given are degrees of freedom (df), *F* values, and *P* values.

For the SNP rs40615, a three‐way interaction was also observed between genotypes, ROI and group (*F*_2.65_ = 108.78, *P* = 0.006, *P* < 0.05, corrected). Further analysis demonstrated an interaction in the cerebellum between genotypes and status (*F*_8.94_ = 35.41, *P* = 0.005, *P* < 0.05, corrected). Post hoc *t‐*test revealed that controls carrying the major allele (T) in rs40615 had higher binding compared to major allele carrying patients (*t* = 3.53, *P* = 0.002, *P* < 0.05, corrected) (Table 5 and Fig. 3b). No difference was detected between minor allele (A) groups, nor between genotypes in patients and HCs (*P* > 0.05).

**Table 5:** Linear mixed model effects summary for the SNP rs40615

	Model rs40615		
Fixed effects	df	*F* value	*P* value
Intercept	14.95	749.56	<.000
group	29.72	0.30	0.59
ROI	86.53	200.42	<.000
rs40615	51.60	0.26	0.62
group*ROI*rs40615	108.78	2.65	0.006
*Separated by ROI*			
Cerebellum group*rs40615	35.41	8.94	0.005
Midbrain group*rs40615	35.94	0.08	0.78
Putamen group*rs40615	35.79	2.05	0.16
Thalamus group*rs40615	34.97	0.46	0.51

Values given are degrees of freedom (df), *F* values, and *P* values

Mean NET BP_ND_ of controls and ADHD group depending on genotype grouping is listed in Table 6. In this relatively small sample, no significant associations between SNPs and ADHD were detected (*P* > 0.05). Highest significance was reached with the rs28386840 SNP for the A allele with a *P* value of 0.09.

**Table 6:** Rounded mean ± SD for NET BP_ND_ values in selected ROIs, shown depending on genotype (major/minor allele) in patients with ADHD and controls

	Controls			
	rs28386840	rs2242446	rs15534	rs40615
ROI	A/T	T/C	C/T	T/A
Putamen	0.16 ± 0.04/0.18 ± 0.04	0.16 ± 0.04/0.19 ± 0.05	0.18 ± 0.04/0.21 ± 0.03	0.19 ± 0.04/0.16 ± 0.02
Midbrain/pons	0.22 ± 0.08/0.30 ± 0.12	0.22 ± 0.08/0.29 ± 0.12	0.26 ± 0.10/0.26 ± 0.11	0.27 ± 0.10/0.25 ± 0.11
Thalamus	0.39 ± 0.07/0.52 ± 0.12	0.39 ± 0.07/0.52 ± 0.13	0.45 ± 0.13/0.48 ± 0.11	0.45 ± 0.13/0.48 ± 0.10
Cerebellum	0.20 ± 0.06/0.26 ± 0.06	0.20 ± 0.06/0.26 ± 0.07	0.24 ± 0.07/0.21 ± 0.06	0.25 ± 0.06/0.20 ± 0.06
	ADHD			
	rs28386840	rs2242446	rs15534	rs40615
ROI	A/T	T/C	C/T	T/A
Putamen	0.18 ± 0.05/0.17 ± 0.05	0.18 ± 0.05/0.17 ± 0.05	0.17 ± 0.03/0.19 ± 0.06	0.17 ± 0.03/0.18 ± 0.06
Midbrain/pons	0.24 ± 0.09/0.22 ± 0.12	0.25 ± 0.10/0.22 ± 0.10	0.25 ± 0.10/0.22 ± 0.11	0.26 ± 0.10/0.22 ± 0.11
Thalamus	0.48 ± 0.08/0.48 ± 0.03	0.48 ± 0.08/0.47 ± 0.04	0.49 ± 0.05/0.47 ± 0.08	0.49 ± 0.05/0.47 ± 0.08
Cerebellum	0.20 ± 0.09/0.21 ± 0.09	0.20 ± 0.09/0.21 ± 0.09	0.17 ± 0.06/0.24 ± 0.10	0.17 ± 0.06/0.23 ± 0.10

### Behavioral Correlation

**Figure 4:**
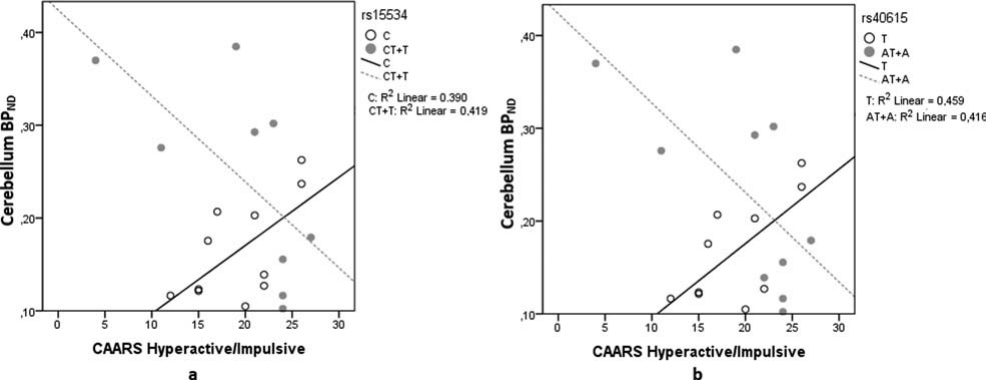
Association between NET BP_ND_ in the cerebellum and the CAARS hyperactive/impulsive scale depending on genotype in rs15534 (*269 T/C) (**a**) and rs40615 (*417 A/T) (**b**). The scatter plot shows the correlation split by major (white circles) and minor (gray circles) alleles in ADHD subjects only. The significance for the SNP rs15534, depending on major allele C was *P* = 0.026, and for minor allele T, *P* = 0.026. For the SNP rs40615, depending on major allele T; *P* = 0.028, for minor allele A, *P* = 0.034.

**Figure 5:**
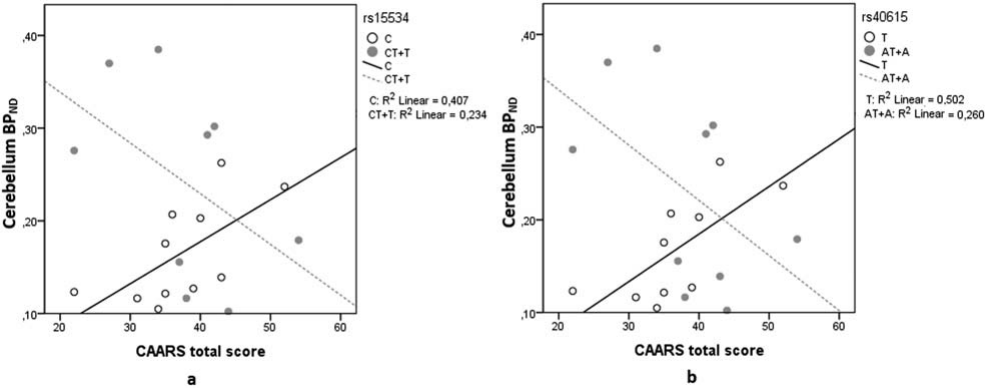
Association between NET BP_ND_ in the cerebellum and the CAARS total score depending on genotype in rs15534 (*269 T/C) (**a**) and rs40615 (*417 A/T) (**b**). The scatter plot shows the correlation split by major (white circles) and minor (gray circles) alleles in ADHD subjects only. Significance was only found depending on the major alleles, for rs15534 (C, *P* = 0.005) and for rs40615 (T, *P* = 0.003).

To test whether these effects were associated with specific ADHD symptoms, scores from CAARS‐Inattentiveness and CAARS hyperactivity/impulsiveness were tested between genotype groups and NET BP_ND_. No correlation of symptoms scores with NET binding was detected in any region in patient groups separated by SNPs rs28386840 and rs2242446 with any region. Conversely, a significant correlation was detected between the behavioral subscales CAARS hyperactivity/impulsiveness (*P* < 0.05) and CAARS total score (*P* < 0.05) with NET BP_ND_ in the cerebellum depending on genotype for rs15534 and rs40615. For the major allele in rs15534, CAARS hyperactivity/impulsivity was positively associated with NET BP_ND_ (*r* = 0.664, *P* = 0.026). For the minor allele group, the scale was negatively associated with NET BP_ND_ (*r* = −729, *P* = 0.026) (Fig. 4a). For the CAARS total score, in the major allele group the positive correlation was *r* = 0.772, *P* = 0.005 (Fig. 5a). No association was detected for the minor allele. For rs40615, differential association between CAARS hyperactivity/impulsivity was also detected depending on genotype. Depending on the major allele, the postive association detected was *r* = 0.689 (*P* = 0.028). On the contrary, the negative association for the minor allele was *r* = −0.669 (*P* = 0.034) (Fig. 4b). For the CAARS total score, the positive association with NET BP_ND_ was *r* = 0.827 (*P* = 0.003) in the major allele group (Fig. 5b). The association for the minor allele did not reach significance.

In addition, for the minor allele group, a negative correlation was detected between NET BP_ND_ in the midbrain with CAARS hyperactivity/impulsivity (*r* = −0,831, *P* = 0.006, rs15534) and (*r* = −0877, *P* = 0.001, rs40615).

## DISCUSSION

Here, we report the influence of genetic variants within the NE system and its effect on in vivo NET binding using PET and the radioligand (*S,S*)*‐*[^18^F]FMeNER‐D_2_. Our results showed significant differences in cerebellar and thalamic NET binding dependent on genotypes between patients with ADHD and HCs. These were largely due to the impact of NET gene polymorphisms on NET BP_ND_ in HCs which is not as pronounced in patients with ADHD. Strikingly, in patients with ADHD, a high correlation between specific behavioural symptoms, that is, hyperactivity/impulsivity, and NET BP_ND_ in the cerebellum was detected, an effect which was strongly moderated by genotype.

Our results for the functional promoter SNP (rs28386840) deviate from in vitro experiments which found that the minor (T) allele resulted in decreased promoter activity and the major allele (A) in higher expression [Kim et al., 2006a]. We detected high NET binding for the minor (T) allele carriers which, indicating high expression of this allele. However, a possible reason for this opposite effect are changes in gene expression based epigenetic mechanisms. The T allele was found to bind to transcriptional repressors, slug, and scratch, which result in decreased expression [Kim et al., 2006b]. Slug recruits a corepressor, which in turn recruits histone deacelytase (HDAC) [Shirley et al., 2010] resulting in tighter packing of the DNA of thus lower transcription of the gene. A counteraction of a repressor, such as degradation, inactivation by interaction with other elements, such as small interfering RNAs or HDAC inhibitors, could lead to overexpression and thus result in a reversed effect of the polymorphism in vivo as observed in our findings [Prelich, 2012; Tuschl, 2001]. Further research is needed to determine the functional effect of the major (A) allele. No significant difference was detected between major and minor allele in ADHD subjects indicating that this effect is not pronounced in ADHD. Moreover, Kim et al. [2006b] found the T allele to be overtransmitted in ADHD, and thus concluded it to be a risk allele for ADHD. Even though no SNP reached significance for association to ADHD in our sample, the strongest effect was seen for the SNP rs28386840 with the A as the associative allele. This is compatible with a recent study by Hohmann et al. [2015] which reports homozygotic A allele carriers to have a higher rate of lifetime ADHD diagnosis. Additionally, another study reported higher response times for ADHD subjects carrying the A allele [Kim et al., 2013]. Nonetheless, one has to bear in mind that studies have been inconsistent, possibly due to confounding factors, such as medication history, individual differences, comorbidities, and differences in sample sizes [de Zubicaray et al., 2008; Leo and Cohen, 2003].

The SNP rs2242446, first determined by Zill et al. [2002] also showed this similar binding in the thalamus as for the functional promoter SNP. This SNP is located on the 5′ flanking region of the NET and the functional effects of the 5′ flanking region is crucial in transcription regulation [Kim et al., 1999; Meyer et al., 1998]. It has been implicated in antidepressant response to milnacipran in depressed subjects. Yoshida et al. [2004] found that major allele (T) carriers responded better to the treatment than the minor (C) allele. The comorbidity of ADHD and depression ranges from 5% to 40%. Symptoms of depression often overlap with those in ADHD, such as distractibility, poor concentration, and impulsivity [Goodman and Thase, 2009; McIntosh et al., 2009]. In addition to the antidepressant effect of milnacipran, it has also been demonstrated to alleviate symptoms of inattention and impulsivity [Hiraide et al., 2013; Kako et al., 2007]. Here, the major allele carrier ADHD group had higher binding than the major allele HC carriers. Thus, the major allele may lend support to the use of milnacipran to treat ADHD patients with comorbid depression.

Noticeable, our major novel findings was the inverse effect of genotype on NET BP_ND_ between controls and patients for rs15534 and rs40615. Even more intriguingly, we detected correlation of scores on CAARS Hyperactivity/Impulsivity and CAARS total score scales with the NET BP_ND_ in the cerebellum for ADHD subject which was strongly modulated by genotype. For both SNPs, in patients carrying the major allele higher NET availability was associated with higher symptom scores. On the contrary, as NET availability decreased, scores increased for the minor allele. From a pharmacological point of view, these findings can only explain the higher NET binding found for the minor allele as it reflects lower NE levels. However, further influencing factors remain unclear. These results resemble the classic inverted U‐shaped effect as Aston–Jones and associates (1999) established for interaction of LC and NE on task performance. The hypothesis states that ADHD symptoms are due to increased tonic activity in the LC which in turn inhibits the basal activity in the cerebellum and other areas and thus increases motor hyperactivity and impulsivity [Berridge and Waterhouse, 2003; Howells et al., 2012]. Aston–Jones and associates research on monkeys showed that with increased tonic discharge of the LC, phasic activity of LC neurons is decreased. This results in poorer performance on focusing on task stimuli. Moreover, they state that for optimal performance, balanced levels of tonic, and phasic activity are needed. If the levels of tonic discharge are too low or too high, attentional performance suffers vastly [Aston‐Jones et al., 1999]. Extracellular levels of NE have been demonstrated to follow a positive linear relationship with tonic discharge from the LC [Berridge and Abercrombie, 1999; Florin‐Lechner et al., 1996]. With that in mind, for the major allele carriers, the tonic release may be too high as NET binding is lower indicating high levels of extracellular NE. This inverted‐U relationship between tonic activity and task performance may explain why we detected this inverse genotype effect. The level of this inverse effect is, therefore, likely determined by the genotype. Along these lines, studies indicate a region specific LC stimulation and LC–NE effect. The LC effect may differ in terms of interaction with receptor subtypes and sensitivity as well as for NE concentration in that region [Berridge and Waterhouse, 2003; Devilbiss et al., 2006]. A study done on healthy rats revealed that tonic stimulation of the LC had differential effects on cortical cells versus cells in the thalamus [Devilbiss and Waterhouse, 2004]. Another study revealed projections to the PFC and the motor cortex to differ [Chandler et al., 2014]. They revealed that neurons projecting from LC to the PFC show more spontaneous activity and are more excitable than those projecting to the motor cortex. The LC might have a differential effect on the thalamus and the cerebellum and this may explain why we detected opposite binding for the major alleles on SNPs located in the 5′UTR versus those in the 3′UTR region.

Another explanation involves the location of these SNPs. They are located in the 5′UTR and 3′UTR regions which have been demonstrated to play an important part in translation, stability and localization of the mRNA. The SNPs r28386840 and rs2242446 are located within the promoter regions while rs40615 and rs15534 are located downstream at the termination codon. The NET may be deregulated by changes in gene expression, mRNA translation or stability, post‐translational modifications such as phosphorylation, protein trafficking, cytoskeleton interaction, and oligomerization [Chatterjee and Pal, 2009]. Substrates involved in the aforementioned processes have also shown to have a regulatory effect on the NET. An injection of the enzyme inhibitor α–methyl‐*p*‐tyrosine (AMPT), resulted in around 50% reduced levels of NE as well as lower mRNA levels in the brainstem indicating a compensatory mechanism for reduced extracellular NE levels [Xiao et al., 1995]. Furthermore, activation of protein kinase C (PKC) has also been shown to regulate the NET. Activation of PKC is believed to result in redistribution of surface NET as radioligand binding demonstrated a reduction in *B*_MAX_ to NET without any change to *K*_D_ [Apparsundaram et al., 1998]. Different location of the SNPs and function may explain why we only detected differences for one allele and why we detected opposite binding on major alleles between SNPs in the 5′UTR and the SNPs in the 3′UTR.

### Limitations and Future Directions

A limitation of this study is that with (*S,S*)*‐*[^18^F]FMeNER‐D_2_ cortical areas cannot be properly assessed due to spill over from suspected bone uptake. Therefore, genetic influence in the neocortex could not be examined. Studying the cortex, specifically the frontal cortex due to its vast role in cognitive and behavioral control would be very intriguing to test whether and what effects polymorphisms in the NET gene would have on the binding. Another limitation is that we did not include the CAARS inconsistency index and, therefore, we could not assess whether there was any inconsistencies or irregularities in responses within subjects, nor could we analyze that in respect to our data.

The evidence presented in this article gives rise to a role for genetic influence of the NE system and alterations in NE signalling as a part of the pathophysiology contributing to ADHD and thus strengthening the hypothesis of imbalances in NE system in the neurobiological mechanism of ADHD. However, we did not detect any differences in the midbrain and the putamen. We can only hypothesize about the possible reasons for these distinct effects. Our results may suggest that the NET genotype effects modulate the NET availability in a region specific manner. Conversely, we cannot exclude other possibilities, such as other influential genetic or nongenetic factors that interact with these regions. In addition, we did not detect any association of any SNP to ADHD, which is probably due to insufficient power of this sample to assess these subtle effects. Due to the heterogeneous nature of ADHD further research requires larger samples for the validation and for the establishment of potential endophenotypes in ADHD. Replication in vivo and in vitro studies is needed to establish if NET genotypic influence could serve as an endophenotype for NE neurotransmission. Moreover, SNPs within the NET may also be very important in relation to the dopaminergic system. The NET is also responsible for reuptake of dopamine in cortical regions [Morón et al., 2002], and therefore, SNPs could possibly affect the availability of dopamine within the cortex. Future studies could explore the possibility whether there are any effects of the NET on the dopaminergic system.

To conclude, this is the first imaging genetic study showing significant differences in NET BP_ND_ in patients with ADHD compared to HCs, depending on their genotype. We find genotypic difference in the thalamus between major and minor alleles for a functional promoter SNP in HCs only. The inverse effect of genotype which was detected in the cerebellum indicates genetic influence of NET on the binding in the cerebellum to differ between groups of ADHD subjects and HCs. The results are compatible with the theory that NE follows an inverted‐U‐shaped curve. Its effect on differential association of behavioral scales with binding further demonstrates a functional and neuropsychological activity to be imbalanced in ADHD.
